# The Role of Lifestyle Interventions in PCOS Management: A Systematic Review

**DOI:** 10.3390/nu17020310

**Published:** 2025-01-16

**Authors:** Rohit Gautam, Pratibha Maan, Anshu Jyoti, Anshu Kumar, Neena Malhotra, Taruna Arora

**Affiliations:** 1 Division of Reproductive Child Health and Nutrition, Indian Council of Medical Research (ICMR), New Delhi 110029, India; rohitgautam.692@gmail.com (R.G.); maanpratibha@gmail.com (P.M.); 2 Department of Obstetrics and Gynecology, All India Institute of Medical Sciences (AIIMS), New Delhi 110029, India; jyotianshu97@gmail.com (A.J.); anshuokkumar@gmail.com (A.K.)

**Keywords:** PCOS, dietary interventions, ketogenic diet, physical activity, lifestyle modifications

## Abstract

Polycystic ovary syndrome (PCOS) is one of the most prevalent endocrine disorders among reproductive-aged women. It is characterized by hyperandrogenism, anovulation, and polycystic ovaries. Lifestyle changes are suggested as first-line interventions in managing PCOS. This systematic review aims to assess the scientific evidence regarding the role of lifestyle modifications (dietary changes, physical activity, and behavioral changes) in improving reproductive, anthropometric, metabolic, and psychological outcomes in women with PCOS. Dietary interventions such as foods with low glycemic index scores; caloric restrictions; high-fiber, omega three fatty acid-rich diets; ketogenic diets; Mediterranean diets; antioxidant-rich food; and anti-inflammatory diets improve insulin sensitivity and hormonal balance in women with PCOS. Physical activity, like aerobic and resistance exercise, enhances insulin sensitivity, helps weight loss, and improves metabolic and reproductive outcomes in women with PCOS. Further, behavioral and education modules can also be used to improve awareness, adherence, and the effectiveness of conventional treatment and to manage mental health issues related to PCOS. Collectively, lifestyle modifications not only improve the biochemical, hormonal, and anthropometric parameters in PCOS patients but also reduce the long-term risks of metabolic and cardiovascular diseases.

## 1. Introduction

**Figure 1:**
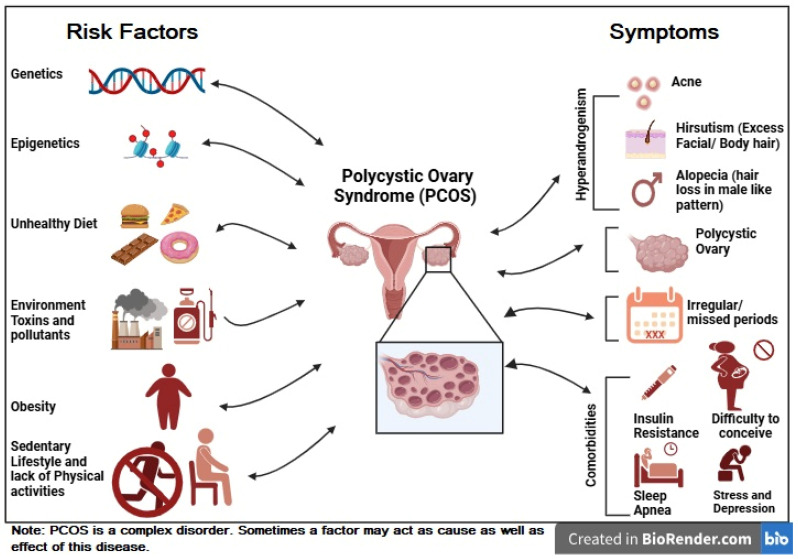
Risk factors and major symptoms of polycystic ovary syndrome (PCOS). The figure was created using Biorender.com.

Polycystic ovary syndrome (PCOS) is a common endocrine disorder affecting women of reproductive age. Globally, it affects around 6% and 20% of women of reproductive age [1,2]. As per Rotterdam criteria, it is characterized by any two symptoms from hyperandrogenism, ovulatory dysfunction (OD, oligo-ovulation/anovulation), and polycystic ovaries (PCO) [3]. Further, PCOS can be categorized into four phenotypes based on the above symptoms. These are phenotype A (OD + hyperandrogenism + PCO), phenotype B (hyperandrogenism + OD), phenotype C (PCO + hyperandrogenism), and phenotype D (OD + PCO) [4]. It is a multifaceted disease with several metabolic disturbances like (insulin resistance), obesity, dyslipidemia, and an increased risk of type 2 diabetes and cardiovascular disease (Figure 1) [5]. One of the main pathophysiological characteristics of PCOS is defined as insulin resistance (IR), which exacerbates the clinical appearance of PCOS and contributes to hyperandrogenism (increase in free androgen levels) [6]. Hyperandrogenism can lead to an increase in PCOS symptoms like acne, hirsutism, and ovulatory dysfunction [7].

Although the exact causes of PCOS are still unknown, it is believed that both endogenous and exogenous factors play a significant role. Various factors like genetics, epigenetics, dietary preference, lifestyle, and environmental factors play an essential role in the etiology of PCOS (Figure 1) [8,9]. Hormonal dysregulation is also one of the hallmark features of PCOS, which manifests in elevated levels of androgens (hyperandrogenism) and the disrupted secretion of gonadotropins like luteinizing hormone (LH) and follicle-stimulating hormone (FSH) [10]. Furthermore, hyperinsulinemia exacerbates hyperandrogenism by stimulating ovarian theca cells to produce excess androgens. This suppresses sex hormone-binding globulin (SHBG), which increases free androgen levels in the [11,12]. Additionally, several immunological factors are recognized as playing roles in the development of PCOS [13]. Immunological factors involved in PCOS pathogenesis include chronic low-grade inflammation, characterized by a rise in IL-6, TNF-α, and CRP levels [14,15]

Oxidative stress and chronic inflammation together play critical roles in the development and progression of PCOS [16]. Oxidative stress exacerbates insulin resistance and hyperandrogenism, while inflammation disrupts ovarian function and increases cardiovascular risk [17]. Because of its multifactorial nature, the management of PCOS is complex, and it requires a comprehensive and individualized approach. According to the most recent international evidence-based guidelines, lifestyle interventions are the primary early strategy for assessing and managing PCOS [18]. Lifestyle changes, dietary patterns, physical activity, and stress management are crucial for controlling the symptoms of PCOS [19].

Diet and exercise are two crucial areas that must be addressed for lifestyle change adjustments to be successful. Dietary habits, including caloric intake, macronutrient composition, and the quality of food choices, play a critical role in weight management, insulin sensitivity, and inflammatory markers in women with PCOS [20]. A diet low in refined carbohydrates and high in fiber can help to regulate blood sugar levels and improve insulin sensitivity. Previous studies have reported the impact of various dietary patterns, including the consumption of low levels of saturated fat, a low glycemic index (GI) score, and the consumption of a high-fiber and ketogenic Mediterranean diet on PCOS management [21,22]. A diet low in refined carbohydrates and high in fiber helps to regulate blood sugar levels and improve insulin sensitivity [23]. Additionally, findings also revealed that dietary supplements such as inositol, vitamin D, and omega-3 fatty acids; mineral supplements (zinc, magnesium selenium, and chromium); and antioxidants like N-acetylcysteine (NAC) help in reducing insulin resistance, improving ovulatory function, and decreasing inflammation in PCOS patients [24,25,26]. Oxidative stress is linked to several clinical conditions, including obesity, type 2 diabetes, and cardiovascular illnesses. An increase in oxidative stress affects the pathophysiology of PCOS patients, which results in insulin resistance, increased androgen, and chronic inflammation [27]. A diet rich in antioxidants or a high-antioxidant-capacity diet (α-tocopherol, vitamin C, vitamin D, polyphenols, and β-carotene) decrease the odds of PCOS [28,29]. Obesity exacerbates PCOS symptoms; therefore, weight loss is also considered one of the strategies of PCOS management. Moreover, dietary modifications can help weight loss in overweight or obese women, lowering IR, testosterone levels, and other risk factors [30].

Physical activity is another cornerstone of PCOS management. Any movement the skeletal muscles produce that requires energy use is considered physical activity [31]. Physical activity and regular exercise (a combination of aerobic and resistance training) help improve insulin sensitivity, reduce body fat, and enhance cardiovascular health in women with PCOS [32]. Exercise also improves psychological wellbeing in women with PCOS. Along with this, various other methods like yoga (breathing, asana, mudras, and meditation), acupuncture, and naturopathy also help in managing regular hormone levels and menstrual cycles and reduce stress and anxiety in women with PCOS [33,34].

Conventional approaches to PCOS treatment have focused on pharmacological interventions like insulin sensitizers, anti-androgens, oral contraceptives, and ovulation induction [35,36]. These methods have not always addressed the underlying lifestyle factors that contribute to the development and persistence of PCOS. Further, this has led to the development of an interest in non-pharmacological approaches, particularly lifestyle modifications, for the management of PCOS. The present review aims to assess the studies in the last ten years and provide a systematic overview of the current evidence regarding the role of lifestyle modifications in managing PCOS.

## 2. Study Selection and Screening

**Figure 2:**
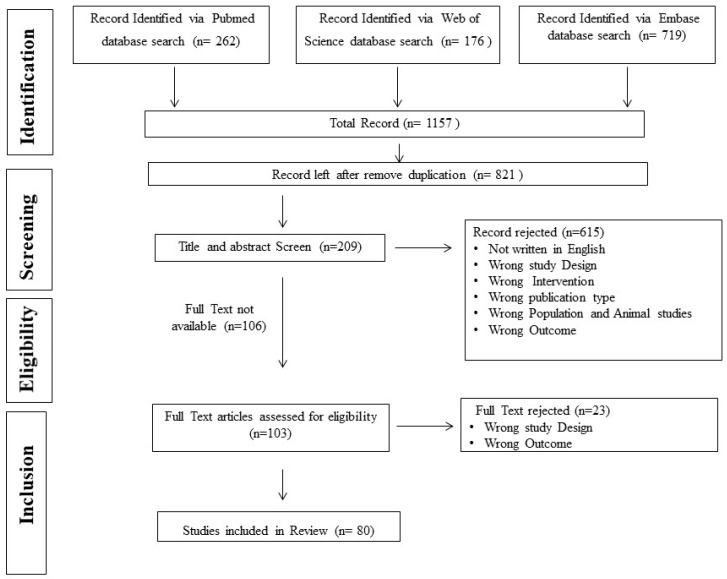
PRISMA flow diagram for screening and selection.

We performed extensive literature searches in the PubMed, Embase, and Web of Sciences databases. The search strategy included the following keywords along with their medical subject headings (MeSH terms): “PCOS” OR “Polycystic ovarian syndrome” OR “Polycystic ovary syndrome” OR “Polycystic ovary disease” OR “Polycystic ovary disorder” OR “PCOD”; “diet” OR “low glycemic index diet” OR “anti-inflammatory diet” OR “high fiber diet” OR “lean protein”OR”omega-3 fatty acids” OR “antioxidant-rich diet” OR “ketogenic diet” OR “keto diet” OR “calorie restricted diet” OR “high protein diet” OR “time restricted diet” OR “microbiome rich diet”; “physical activity” OR “aerobic exercise” OR “regular exercise” OR “resistance training” OR “yoga” OR “meditation” OR “lifestyle” OR “quality of life”; “behavior modification” OR “mindfulness training program” OR “education module”; “clinical trial” OR “randomized controlled trial”. Only articles written in the English language and published in the last 10 years were included (Figure 2). Studies conducted in animal models and in vitro models were excluded. Only randomized controlled trials were included and all other study designs, such as observational studies, single-arm studies, systematic reviews, narrative reviews, etc., were excluded. Two authors screened titles and abstracts to verify their inclusion using Rayyan software (https://new.rayyan.ai/). Additional references from included studies were also searched wherever necessary.

We have received a total of 1157 studies, out of which 555 studies were found to be duplicates. After removing duplicates, 821 studies were primarily screened by title and abstract. After primary screening, 209 articles were selected, but only 103 articles in the full text were retrieved. After full-text screening, 80 papers were finally included in the study. Further papers were divided into three categories: ‘diet and supplements’, ‘physical activity’, and ‘behavioral and education model therapy’. This review was further developed by addressing each subcategory separately.

## 3. Dietary Habits and Supplements in PCOS Management

**Figure 3:**
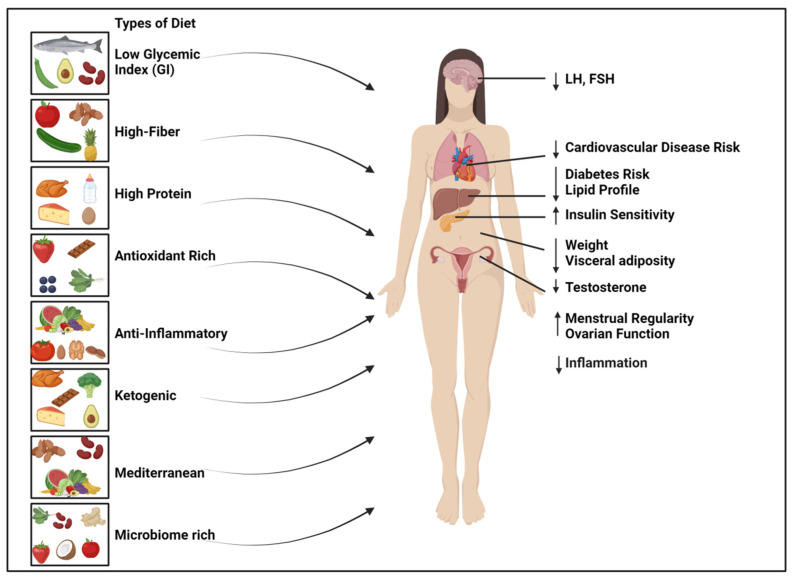
Effect of different dietary patterns on PCOS symptoms reduction. ↑ Sign indicate increase and ↓ sign indicate decrease in the symptoms mentioned adjacent to it. Figure was created using Biorender.com.

Several types of diets were assessed for PCOS management (Figure 3). The results of such RCT studies are summarized in Table 1. Each type of diet and its mechanisms are summarized below.

### 3.1. Low-Glycemic-Index (GI) Diet

A low-GI diet includes foods that cause a slow, gradual rise in blood sugar levels, helping to improve insulin sensitivity [86]. Further, stable insulin levels help to reduce excess androgen (testosterone) production and help to control symptoms like acne, hirsutism, and irregular menstrual cycles [87]. Studies have shown that incorporating a low-GI diet can play a significant role in controlling the metabolic and hormonal imbalances associated with PCOS in women. Additionally, low-GI foods, like whole grains (oats, quinoa, barley), legumes (beans, lentils), fruits (berries, apples, pears), and vegetables (leafy greens, broccoli, cauliflower), are rich in anti-inflammatory compounds. Findings show that dietary intervention (via a low-calorie, low-glycemic diet) increases uric acid and GPx3 (glutathione peroxidase) activity, further reducing inflammation and oxidative stress in women with PCOS [88]. In an RCT study on 37 women with PCOS, low-glycemic-index diets were given to 19 women, and 18 women received diets with normal glycemic index scores. The results showed an increase in ovulatory cycles and improvement in insulin resistance with decreased serum androgen levels [89]. In recent meta-analysis findings, low-GI diets reduce HOMA-IR, fasting insulin, total cholesterol, LDL cholesterol, triglycerides, waist circumference, and total testosterone levels compared to HGI diets in women with PCOS [90].

### 3.2. Ketogenic Diet

The ketogenic diet (KD) is a high-fat, adequate-protein and low-carbohydrate diet that forces the body into a metabolic state called ketosis [91]. In this condition, the body becomes highly adept at using fat rather than carbohydrates as fuel [92]. A ketogenic diet contains fats (55–60%), proteins (35%), and carbohydrates (5–10%) in total daily calories [93]. The therapeutic role of the KD diet has been observed in diabetes, obesity, epilepsy, depression, renal function, Alzheimer’s cardiovascular disease, etc. [94,95]. In an umbrella review including 17 meta-analyses of 68 RCT results, VLCKD (very-low-calorie ketogenic diet) was significantly associated with improving anthropometric and cardiometabolic outcomes [96]. Women with PCOS are frequently obese or overweight, which exacerbates IR and hormone abnormalities. The ketogenic diet may result in more successful weight loss because the body burns fat instead of glucose when in the metabolic state of ketosis. Studies have reported that a ketogenic diet helps to lower androgen levels and improve reproductive hormone levels, insulin sensitivity, and SHBG levels in PCOS women [21]. Similar results have been observed in a systematic review and analysis performed on 11 RCTs: the findings showed that the ketogenic diet significantly decreases [96] weight loss, BMI, waist circumference, and fat mass in obese or overweight women with PCOS [97]. However, it cannot be recommended in the long term as it has low carbohydrate and high fat content which can negatively affects the body as it has less nutritional value. Most of the studies have also intervened in KD for a shorter period only, with gradually increasing the calories during the intervention.

### 3.3. Anti-Inflammatory Rich Diet

Systemic inflammation plays a role in the etiology of PCOS [98]. In obese people, adipocytes release many pro-inflammatory cytokines that activate macrophages, leading to chronic low-grade inflammation. Further, increased cytokine levels result in disorder progressions like cardiovascular disease and insulin resistance [99,100]. Anti-inflammatory diets, rich in fruits, vegetables, whole grains, and omega-3 fatty acids, can help to mitigate inflammation. In RCT, the beneficial effects of green cardamom on the inflammatory markers in women with PCOS were observed. Findings from the study showed that inflammatory markers like TNF-α and IL-6 and CRP serum levels significantly declined in the intervention group compared to the placebo group [43]. Moreover, similar results were observed in a pilot study by Mizger et al., which showed that an anti-inflammatory diet improves total antioxidant capacity, IL-1 and IL-6, TNF-α, and androstenedione levels in girls with PCOS [101]. In addition to the above foods, onion (*Allium cepa* L.) has good anti-inflammatory properties, and results from clinical trials studies revealed that obese/overweight women with PCOS who consume a high dose of raw red onion showed a decrease in serum adiponectin, leptin, and hs-CRP level with non-significant changes in IR and anthropometric parameters [102,103].

### 3.4. Antioxidant-Rich Diet

Oxidative stress is an imbalance between antioxidants and oxidants in living biological systems [104]. Oxidative stress plays a vital role in the pathogenesis of PCOS, leading to insulin resistance, androgen excesses, and chronic inflammation [105,106]. Women with PCOS have an imbalance in their total serum antioxidant content, which exacerbates cellular damage and alters the defense mechanism [107]. Studies have shown that pregnant women with PCOS have high serum MDA (malondialdehyde) levels (an oxidative stress marker) and low antioxidant vitamin levels compared to healthy pregnant women [108,109]. Vitamin D plays a significant role as an antioxidant, reducing oxidative stress and inflammation in the body, and it helps to mitigate the harmful effects of reactive oxygen species (ROS) [110]. In a double-blind, placebo-controlled trial on 70 vitamin-deficient women with PCOS, when administrated to vitamin D supplementation for 4 weeks, the results showed beneficial effects on glucose homeostasis parameters, hs-CRP, and MDA levels [111]. Further findings from various clinical trials have shown that vitamin D supplementation to women with PCOS help to improve insulin sensitivity, glucose metabolism, lipid profiles, HOMA-B (homeostasis model of assessment-estimated B cell function), adiponectin, and other biochemical parameters [112,113,114]. In another RCT, women with PCOS, co-administered with vitamin D and omega-3 fatty acid for 12 weeks, saw beneficial effects on serum total testosterone, hs-CRP, and plasma TAC and MDA levels [115]. In addition to the above-mentioned antioxidant food, clinical trials have shown that the administration of various antioxidant supplements like selenium, green cardamom, astaxanthin, berberine phytosome, and isoflavones helps to improve anthropometric parameters, biochemical, hormonal and metabolic symptoms in women with PCOS [116,117,118,119,120].

### 3.5. Omega-3 Fatty Acids Rich Diet

Omega-3 fatty acids have powerful anti-inflammatory properties that can improve insulin sensitivity and decrease androgen levels in metabolic syndromes like PCOS [121]. An increasing intake of omega-3 and other PUFAs can help to reduce and alleviate low-grade chronic inflammation and other symptoms in women with PCOS via TGF-β signaling [122]. The sources of omega-3 fatty acids are fish, like salmon, mackerel, sardines, trout, and nuts, and seed oils, like flaxseed oil, chia seed oil, and walnut oil. The results from a double-blind RCT conducted on 64 overweight or obese women with PCOS showed improvement in serum adiponectin levels, IR, and the lipid profile in the intervention group as compared to the control group [123]. Studies also showed that omega-3 and vitamin E intake effectively improved the mental health parameters and gene expression of PPAR-γ, IL-8 and TNF-α in women with PCOS [124]. Further, omega-3 fatty acid supplements in women with PCOS helped to improve biochemical parameters LH, LH/FSH, lipid profiles, WC, adiponectin levels, and regularized the menstrual cycle [125,126].

### 3.6. High-Fiber, High-Protein Diet and Other Dietary Supplements

Foods rich in fiber content and a high-protein diet, preferably from lean protein sources, helped to slow the absorption of sugar into the bloodstream, preventing spikes in blood glucose and insulin levels [127]. Improved insulin sensitivity can help to reduce androgen levels and improve androgen levels, hormones, and menstrual irregularities. High-fiber foods include vegetables (broccoli, spinach, carrots, and kale), fruits (apples, berries, pears, and oranges), whole grains (brown rice, quinoa, whole oats, and barley), legumes (lentils, chickpeas, and black beans), and nuts and seeds (almonds, flaxseeds, and chia seeds). Studies have shown that fiber intake in women with PCOS was negatively correlated with IR, fasting insulin, glucose tolerance, and androgen levels [128]. Additional findings from an RCT study on 48 women with PCOS showed a reduction in serum insulin, TAG and LDL, and cholesterol and a significant increase in TAC and GSH levels in those groups in which women consumed a diet rich in fruits, vegetables, whole grains, and low-fat dairy products. Many dietary supplements like carnitine reduce carotid intima-media thickness and inflammatory factors in women with PCOS [129]. The administration of melatonin in women with PCOS also showed a reduction in hirsutism, testosterone, hs-CRP, and MDA levels with an elevation in TAC and GSH levels [130].

In addition to the above-mentioned dietary supplements DASH (The Dietary Approaches to Stop Hypertension) diet is also found be effective for women with PCOS, improving reproductive health [131]. A DASH diet is rich in fruits, vegetables, whole grains, and low-fat dairy products and low in saturated fats, cholesterol, refined grains, and sweets; in addition to this, the intake of sodium should be less than 2400 mg/day [132]. The DASH eating plan is a low-glycemic-index low-energy-dense diet that was first suggested to lower blood pressure [133]; however, the beneficial effects of DAH diet alone and with whole-food, plant-based diets have also been reported regarding insulin resistance, inflammation diabetes, and metabolic syndrome [134,135,136].

### 3.7. Microbiome Rich Diet

Research has shown the existence of a close association between the gut microbiota of PCOS patients and the development of insulin resistance, which is a corresponding factor for PCOS, manifesting through short-chain fatty acids, lipopolysaccharides, sex hormones, and the brain–gut axis [78]. Compared with healthy people, the diversity in the gut microbiota of PCOS patients shows a reduction with changes in composition, damaging the intestinal mucosal barrier [137]. Healthy gut microbiota can be restored by switching to a healthy diet. A microbiome-rich diet is rich in fermented foods, whole grains, fruits/seeds, and fiber. It can also be supplied via probiotic or symbiotic supplements. In an RCT where women with PCOS were treated with multispecies probiotic supplementation for 8 weeks, the results showed non-significant improvement in pancreatic β-cell function and the CRP level in women with PCOS [138]. In another double-blind RCT on sixty women with PCOS, the administration of synbiotic supplementation (Lactobacillus and Bifidobacterium strains) for 12 weeks resulted in beneficial effects on IR, triglycerides, and VLDL cholesterol levels [116]. A meta-analysis of 7 RCTs revealed that probiotic/symbiotic supplementation decreased insulin and triglyceride levels and increased HDL levels compared to the control in women with PCOS. Although insignificant, they also improved other biochemical and anthropometric parameters [139]. Another meta-analysis of 8 RCTs showed that probiotic/symbiotic treatment can effectively improve hormonal, glucose homeostasis, and inflammatory outcomes of PCOS [140]. Therefore, they can also serve as a good option for PCOS management.

## 4. Physical Activity (Aerobic Exercise and Resistance Training) and PCOS

**Figure 4:**
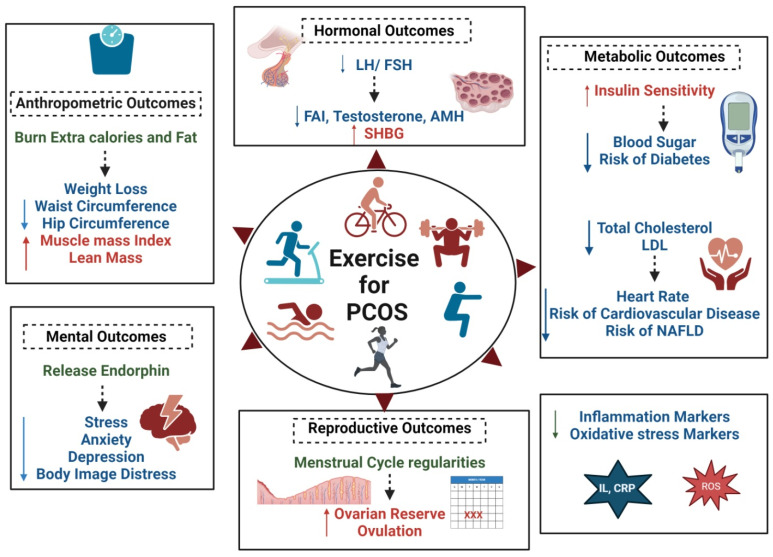
Impact of physical activity/exercise on anthropometric, hormonal, metabolic, reproductive, and mental health outcomes in women with PCOS. ↑ Sign indicate increase and ↓ sign indicate decrease in the outcome mentioned adjacent to it. Figure created using Biorender.com.

Aerobic exercise is any activity that uses large muscle groups, is maintained continuously, and is rhythmic in nature [141]. Studies have proven that exercise, particularly aerobic workouts, offers multiple benefits for managing PCOS symptoms, such as improving insulin sensitivity, cardiovascular health, and hormonal balance (Figure 4) [142,143]. Aerobic exercise, such as brisk walking, running, cycling, or swimming, helps to enhance insulin sensitivity by improving how muscles absorb glucose from the blood. It may regulate menstrual periods by lowering insulin levels and enhancing ovarian function [144,145]. In a study on women with PCOS, a significant decrease in BMI and fat mass was observed, along with an increase in FSH, free testosterone, and sex-hormone-binding globulin (SHBG) levels, in the experimental group (exercised for 12 weeks, 3 sessions of 20 min) as compared to the control [146]. International guidelines for the management of PCOS also recommended that adults (18–64 years) should aim for a minimum of 250 min/per week of moderate-intensity activities or 150 min/per week of vigorous-intensity activities or an equivalent combination of both, plus muscle-strengthening activities (e.g., resistance/flexibility), ideally on two non-consecutive days per week [18]. Various types of aerobic exercises which can be used to improve PCOS symptoms are listed below:

(i) HIIT (high-intensity interval training) includes short bursts of intense activity followed by rest or low-intensity periods. (ii) MICT (moderate-intensity interval) training includes moderate-intensity exercise, such as jogging, swimming, or cycling. (iii) IAT (intermittent aerobic training) is similar to HIIT but has lower intensity and longer active intervals. (iv) CAT (continuous aerobic training) includes steady-state aerobic exercise with consistent pace and intensity and long-distance running or cycling. The results of clinical trials that assess the impact of physical activity in managing PCOS are summarized in Table 2.

Resistance training is a kind of strength or weight training encompassing workouts that enhance muscle strength and endurance by working against some form of resistance. Findings from various studies have shown that resistance training alone and along with aerobic exercise helped in reducing the symptoms of PCOS. Combined (RT resistance training + MICT) is a type of physical exercise that incorporates both aerobic (moderate-intensity) and resistance (strength) training and also helps to improve metabolic function, overall fitness, and weight management in women with PCOS. A systematic review including 7 RCTs showed that physical exercise helped to improve reproductive functions, hormonal balance, and menstrual cycle regularity [160]. Likewise, meta-analysis related to physical exercise interventions on women with PCOS (data from 19 studies) has shown that physical exercise helps to reduce BMI and waist circumference. Results also suggested that a minimum of 120 min per week of vigorous-intensity exercise is required to produce beneficial health effects for women with PCOS [161].

## 5. Behavioral and Education Module

A lot of mental issues, like depression, anxiety, body image concerns, low self-esteem, eating disorders, stress, emotional strain, mood swings, sleep disorders, etc., are also associated with PCOS [162]. These issues often degrade patients’ quality of life and must be addressed via conventional treatment [163,164]. Developing a behavioral or education model can be an effective way to help women with PCOS to improve the effectiveness of conventional treatment and provide a holistic approach to PCOS management [165,166]. These modules may include education about understanding PCOS and treatment options available, behavioral interventions (diet, exercise, sleep), stress management (relaxation techniques, cognitive therapy), dealing with body changes, goal setting, tracking progress, positive reinforcement, etc. [167,168]. They can be designed as counselling/therapy sessions, group discussions, workshops, education materials, or mobile apps [169,170]. Table 3 summarizes the results of RCTs that have studied the effectiveness of these modules in PCOS patients.

In an RCT involving 161 patients, a one-time structured education module significantly (*p* < 0.05) improved the understanding of PCOS and quality of life (emotions, fertility, weight and mental wellbeing), but could not improve anthropometric and biochemical parameters [182]. Another study focused on 181 women with PCOS who wanted to conceive and received 20 group sessions of cognitive behavioral therapy (CBT) with or without a short message service (SMS) as compared to standard care. Groups receiving CBT showed better weight loss results than those receiving standard care, and the inclusion of SMS further enhanced the benefits [183]. Additionally, an RCT study with 28 women with PCOS was administered with 2 months of mindfulness stress management training, which resulted in a significant decrease in anxiety, depression, and stress and an increase in quality of life as compared to the control [184]. In a recent meta-analysis (8 RCTs), it was shown that supplementing behavioral interventions to routine treatment of PCOS plan enhances the reduction in weight, BMI, and waist circumference and improvement in depression conditions [185]. In a meta-analysis of 8 studies, it was observed that cognitive behavioral therapy helps to reducing anxiety and improve the quality of life, compliance, and pregnancy rate in PCOS patients [186].

Further, several clinical trials have been conducted using multiple lifestyle interventions like dietary measures, behavioral measures, and physical exercise for the treatment of PCOS. In a randomized three-arm parallel study, women with PCOS received structured support, including dietary counseling, a tailored exercise regimen, and behavior change strategies to improve physical activity levels. Results showed that BMI, waist circumference, and total cholesterol levels were significantly decreased in the intervention group. Findings from the study showed that dietary changes were associated with better metabolic outcomes, suggesting that targeted nutritional adjustments are essential for effective PCOS management, particularly for weight control and reducing metabolic risks [187]. In an RCT, women with PCOS received a structured lifestyle program focused on diet and exercise. This was tailored to improving physical activity levels and addressing common exercise barriers. The intervention significantly reduced perceived barriers to exercise. It enhanced perceived benefits, suggesting that lifestyle modification can make exercise more accessible and appealing for women with PCOS, potentially supporting better long-term health outcomes [151].

## 6. Future Perspective

As research on PCOS treatment goes on, lifestyle modifications like dietary changes, physical activity, and behavioral changes are recognized as essential components of its management or treatment. Integrating multiple lifestyle modifications provides the most comprehensive benefits in managing PCOS. Evidence shows that these changes help to improve PCOS symptoms like insulin sensitivity, weight management, menstrual cycle regularity, and metabolic parameters. Further, due to heterogeneity in PCOS in terms of phenotypes, clinical presentations, underlined pathophysiology, and response to treatment, personalized interventions are needed. This can be achieved using artificial intelligence to plan individualized dietary/exercise interventions [188]. Further, women with PCOS often experience depression, anxiety, and a diminished quality of life, which can undermine adherence to lifestyle changes. Future research should explore integrative approaches that combine lifestyle interventions with mental health support, such as cognitive-behavioral therapy or mindfulness/meditation practices, which can reduce depression and improve quality of life.

## 7. Conclusions

In conclusion, lifestyle modifications, encompassing dietary changes, physical activity, and behavioral interventions, are pivotal in managing PCOS by addressing both metabolic and reproductive complications. Evidence from various RCTs suggests that dietary, interventions such as low-glycemic-index foods; caloric restrictions; high-fiber, ketogenic, and Mediterranean diets; omega three- and antioxidant-rich food; and anti-inflammatory diets help to improve insulin sensitivity and hormonal balance and reduce PCOS symptoms. Out of these, the DASH diet and low-glycemic-index (GI) diet are highly effective for PCOS management as they improve insulin sensitivity, reduce androgen levels, and regulate menstrual cycles. Likewise, regular physical activities (aerobic and resistance exercises) also enhance metabolic function and help to reduce weight, achieve glucose homeostasis, balance hormone levels, and improve overall quality of life. Among all the forms of exercise, the aerobic exercise (AE) was found to be more effective in PCOS. Furthermore, continuous aerobic training (CAT) and intermittent aerobic training (IAT) were found to be equally effective in reducing anthropometric indices and hyperandrogenism and in the improvement of quality of life (QOL) in women with PCOS. Further, the future of PCOS management lies in integrating personalized dietary and physical exercise interventions using technology-driven approaches.

## Figures and Tables

**Table 1:** List of studies (RCTs) showing the effects of different dietary and supplement interventions on women with PCOS.

S. No.	Participant Details (*n* = Sample Size)	Location	Study Design	Intervention and Duration	Outcomes Studied	Major Findings *	Reference
**Low-Glycemic-Index (GI) Diet**							
1.	Women with PCOS, BMI < 45 kg/m^2^, aged 21–50 years (*n* = 30)	Birmingham, UK	Crossover RCT	1 = LGL diet (41/19/40%, cho/protein/fat) 2 = HGL diet (55/18/27% cho/protein/fat) 8 weeks	Self-reported appetite, ghrelin, glucagon	↑ 4 h glucagon (1) ↓ Ghrelin (1) ↑ Appetite score in early PP phase (2)	[37]
2.	Women with PCOS, (13–21 years), BMI > 85th percentile (*n* = 19)	Boston, US	RCT	1 = LGL (45/20/35%, cho/protein/fat) 2 = LF (55/20/25%, cho/protein/fat) 6 months	HT, WT, BMI, FT, TT, SHBG, DHEAS, TC, LDL, HDL, TG, hs-CRP, progesterone, OGTT, INS, HRQoL	↓ BMI (2) > (1) ↓ BF, trunk fat (1 = 2) ↓ TC/HDL (1)	[38]
**Ketogenic Diet**							
3.	Women with PCOS, aged (18–45 years), BMI 28–40 kg/m^2^ (*n* = 22)	Bologna, Italy	RCT open label	1 = VLCKD (Step 1 = 600/800 kcal with protein, <50% carb, 10 g olive oil, Step 2 = adding 1 portion natural protein in 1 meal, Step 3 = adding 2 portion of natural protein) Plus vitamin and mineral supplement 2= LCD (1200/1420 kcal–15/30/55% protein/lipids/cho) 16 weeks	LH, FSH, E2, SHBG, HT, WT, BMI, WC, HC, FM, FFM, FG score, INSF, HDL, IR, TC, DBP, SBP, HOMA IR	↓ BMI, WC, FM, FFM, DBP, SBP, INSF, HOMA IR, TC, HDL (1)	[39]
4.	Women with PCOS, aged (18–45 years), BMI > 25 kg/m^2^ (*n* = 14)	Padova, Vicenza territory	RCT (single-arm study)	1 = KEMEPHY, (1600/1700 kcal with phytoextracts) 12 weeks	WT, BMI, FBM, LBM, VAT, FBM%, HDL, LDH, TG, TC, FT, TT, HOMA IR, INS, BG, E2, LH, FSH, DHEAS, LH/FSH, SHBG, FG score	↓, WT, BMI, FBM, VAT, TG, TC, LDL, FBG, INS, LH, FSH, LH/FSH, FT ↑ HOMA IR, HDL, SHBG, E2, progestrone	[40]
**Antioxidant-rich diet and omega-3 supplement**							
5.	Women with PCOS, aged 18–45 years, (*n* = 41)	Iran	RCT open label	1 = lifestyle modification + flax seeds (30 G) 2 = lifestyle modification 12 weeks	HT, WT, BMI, WC, INS, HOMA IR, QUICKI, TT, SHBG, FAI, FG score, hs-CRP, IL-6, adiponectin, leptin, TG, BG, HDL, LDL, TC	↓ WT, BMI, (1 = 2) ↓ WC, FBG, hs-CRP, FAI, INS, HOMA IR, TG, LEPTIN (1) ↑ QUICKI, HDL, adiponetin, menstrual regularity (1)	[41]
6.	Women with PCOS, aged (18–40 years), BMI > 25 kg/m^2^ (*n* = 62)	Iran	Double-blinded RCT	1 = 2 omega-3 pills/day (180 mg EPA + 120 mg DHA) + 400 IU vitamin E 2 = oral paraffin (placebo) 8 Weeks	TAC, GSH, CAT, MDA, DIET, PAL	↑ TAC, CAT (1) ↓ MDA (1)	[42]
7.	Women with PCOS, aged (18–45 years), BMI > 30 kg/m^2^ (*n* = 199)	Iran	RCT (double-blinded)	1 = 3 g cardamom/meal +LCD 2 = LCD + placebo (3 capsule starch powder) 16 weeks	WT, BMI, WC, FM, LH, FSH, TSH, PRL, TT, AD, DHEAS, TNF-α, IL-6r, hs-CRP	↓ WT, BMI, WC, FM (1 = 2) ↓ AD, DHEAS, LH, FSH, TNF-α, IL-6, CRP (1)	[43]
8.	Non-diabetic PCOS women, aged > 18 years, (*n* = 96)	Spain	Partial RCT	1 = MN group (ALA, 75 mg + NAC,100 mg + B_6_, 0.65 mg + SAMe, 200 mg) 2 = OC group (Ethinylestradiol 0.02 mg + Drospirenone 3 mg) 3 = MN + OC group 6 months	BMI, WT, INS, BG, TG, TC, HDL, LDL, ALT, AST, GGT, BP, BIL, HOMA IR,17βEstradiol, 17 OHP, LH, FSH, FT, SHBG, DHEAS, AD, acne, hirsutism, alopecia, infertility, and QoL	↓ HOMA-IR (1) ↓ 17β-estradiol, LH, LH/FSH (2 = 3) ↓ SHBG (2) ↓ DHEAS (3) ↓ AD, acne, hirsutism, QoL, menstrual irregularity (1 = 2 = 3) ↑ QoL (1 = 2 = 3)	[44]
9.	PCOS women, aged (18–45 years), BMI < 30 kg/m^2^ (*n* = 60)	Iran	RCT (double blind)	1 = 1 cap (200 mg EA) 2 = placebo 8 weeks	WT, BMI, FBG, INS, IR, TC, TG, LDL, HDL, TAC, CRP, MDA, TNF-α, AMH, LH, FSH	↓ FBG, INS, IR, TC, TG, LDL, MDA, CRP, TNF-α, TT, PRL, AMH (1) ↑ TAC	[45]
10.	PCOS infertile women, aged (25–38 years) (*n* = 34)	Jerusalem	RCT (double blind)	1 = omega-3 supplementation (3 × 600 mg) (360 mg EPA and 240 DHA) 2 = placebo (sunflower oil)	HT, WT, BMI, LH, FSH, TT, ET, follicle number, E2	26.7% clinical pregnancy with ↓ BMI (1) 13.3% clinical pregnancy (2)	[46]
11.	PCOS women, aged (20–45 years) BMI 30–40 kg/m^2^ (taking OCPs) (*n* = 48)	Iran	RCT (double blind)	1 = Thylakoid-rich (5 g spinach extract) + hypocaloric diet 2 = placebo (5 g cornstarch) + hypocaloric diet 12 weeks	HT, WT, BMI, HC, WC, LPF, BNDF, OS (MDA, TAC and CAT), S 100B, LH/FSH, FBG, HOMA IR, FTI, INS, PAL	↓ LPF (1) ↑ BNDF (1) ↓ FBG, HOMA IR, LH/FSH, FTI (1 = 2)	[47]
12.	PCOS women, aged 18–45 years, (*n* = 80)	India	RCT	1 = intervention (*n* = 40), NAC—1200 mg/day, twice/day 2 = control (*n* = 40), metformin—1500 mg/day, twice/day 6 months	HOMA IR, WT, QoL, BMI, AD, TT, SHBG, menstrual regularity	↓ HOMA IR, TT, (1 = 2) ↓ AD (1) ↑ QoL (1)	[48]
13.	PCOS women, aged 20–50, BMI 30–30 kg/m^2^ (*n* = 80)	Iran	RCT	1 = INTERVENTION (*n* = 40), 800 mg/day garlic pill 2 = CONTROL (*n* = 40), starch 8 WEEKS	WT, BMI, WC, HC, WHR, TAC, CAT, GSH, MDA	↓ WT, BMI, WC, CAT, GSH	[49]
14.	PCOS women, aged 20–40 years, BMI 25–35 kg/m^2^ (*n* = 86)	Iran	RCT	1 = 200 mg CoQ10 daily + vitamin E placebo (*n* = 22) 2 = 400 IU vitamin E daily plus CoQ10 placebo (*n* = 22) 3 = 200 mg CoQ10 plus 400 IU vitamin E daily (*n* = 21) 4 = CoQ10 placebo plus vitamin E placebo (*n* = 21) 8 weeks	WT, BMI, LH, FSH, TT, SHBG, P, E2, FAI, HOMA IR	↓ TT, FBG (1 = 2 = 3) ↑ SHBG (3) ↓ HOMA IR (1 = 3)	[50]
15.	PCOS women, aged 18–40 years, (*n* = 80)	Iran	RCT	1 = intervention (*n* = 40) oligopin 50 mg 2 = control (*n* = 40), maltodextrin 3 months	WT, BMI, WC, TT, FAI, SHBG, DHEAS, LH, FSH, HbA1C, HOMA IR, FG score, PRL, C-Peptide, INS, TSH, FBG, ALT, AST, ALP, TG, TC, HDL, LDL, BUN, hs-CRP	↑ FSH (1)	[51]
16.	PCOS women, aged 18–45 years, (*n* = 72)	Iran (Fatty Acid)	RCT (double blind)	1 = intervention canola (25 mg) (*n* = 24) 2 = intervention olive (25 mg) (*n* = 24) 3 = control sunflower (25 mg) (*n* = 24) (45–60% CHO, 30–35% fat, 15–18% protein) 10 weeks	WT, BMI, TG, HDL, LDL, TC, SHBG, FBG, INSF, FATTY LIVER GRADE, MUFA	↑ MUFA (1 = 2) ↓ HOMA IR (1 = 2 > 3), ↓ FATTY LIVER SEVERITY (1 > 2) ↓ TG, TG/HDL, TC/HDL, LDL/HDL, TC, LDL, (1)	[52]
17.	PCOS women, aged 19–35 years, (*n* = 60)	Viena, Austria	RCT (double blind)	1 = MSG (1 Cap omega 3 fatty acid + 1 Cap FA, Vit E, Se, catechin, glycyrrhizin, co-enzyme Q10 2 = 2 capsule(200 μg FA each) 3 months	AMH, TT, AD, LH, FSH, LH/FSH, SHBG, E2, TSH, PRL,	↓ LH/FSH, AMH, TT (1)	[53]
**Vitamin D Supplement**							
18.	Vitamin D-deficient PCOS women, aged 21–34 years (*n* = 60)	China	RCT	1 = Intervention (*n* = 30), vitamin D 2000 IU/day + Healthy diet + physical activity 2 = control (*n* = 30), regular treatment 12 weeks	WT, BMI, WC, HC, WHR, AMH, LH, FSH, PRL, E2, P, TT, OGTT, HOMA IR, TC, TG, LDL, HDL, INS	↓ BMI, WHR, INS, HOMA IR, TG, TC, LDL (1)	[54]
19.	PCOS women, aged 18–45 years, BMI 18.5–40 kg/m^2^ (*n* = 80)	Iran	RCT	1 = Vit D (*n* = 20), vitamin D 50,000 IU/weekly + placebo (paraffin oil daily) 2 = O3 (*n* = 20), 2 omega-3 capsule (360 mg ESA + 240 mg DHA) + placebo 3 = Vit D + O3 (*n* = 20), vitamin D 50,000 IU + 2 omega-3 capsule 4 = placebo (*n* = 20), paraffin oil 8 weeks	WT, BMI, WC, TG, TC, LDL, HDL, FBG, HOMA IR, SHBG, PAL, INS	↓ WC (1) = (2) = (3) ↓TG, (2) = (3) > (1) ↓TC, (3) > (2) > (1) ↓ FBG (3) > (1) = (2) , ↓ INS, (3) > (2) > (1) ↓ HOMA IR, [(1 > 2) = 3] ↑ SHBG [(3 > 1) = (2)]	[55]
**High-Protein and High-Fiber Diet**							
20.	PCOS women (*n* = 64)	Iran	RCT (double blind)	1 = HSDF (hypocaloric) std diet, 55/15/30% carb/protein/fat + fennel, 2 cap/day) 2 = HHPF (hypocaloric high-protein diet, 40/30/30% carb/protein/fat + fennel, 2 capsule/day) 3 = HSDP (hypocaloric std diet + placebo) 4 = HHPP (hypocaloric high-protein diet + placebo) 3 month	HT, WT, BMI, WC, HC, WHR, TT, SHBG, FAI	↑ Protein intake (2) (did not meet the daily recommendations for protein)	[56]
21.	PCOS women, aged 18–40 years, (*n* = 60), stratified by BMI < 25 kg/m^2^, >25 kg/m^2^)	Iran (protein)	RCT	1 = test diet, (*n* = 30)–0.8 g protein/kg/body weight (35% animal proteins, 35% textured soy protein and 30% vegetable proteins) 2 = control, (*n* = 30)–70% animal proteins including meats, poultry, fish and dairy, and 30% vegetable proteins including grains and vegetables 8 weeks	WT, BMI, WC, HC, INSF, HOMA IR, QUICKI, FG score, menstrual irregularities, FBG, TG, HDL, LDL, VLDL, SFA, hs-CRP, NO, TAC, GSH, MDA, LH, FSH, TT,	↓ WT, BMI, WC, HC, SFA (INTAKE), INSF, HOMA IR, FBG, TT, TG, VLDL, MDA (1) ↑ QUICKI, NO, GSH (1)	[57]
22.	PCOS women, aged 15–41 years, (*n* = 25)	China (high-protein)	RCT	1 = WTP (*n* = 14), high-fiber diet + prebiotic whole grains 2 = A (*n* = 11) (WTP + acarbose 50 mg 3 times/day) 12 Weeks	WT, BMI, OGTT, HbA1C, TT, FG score, LH/FSH, LH, FSH, LIPIDS, OREXIN, SPEXIN, IR, LEPTIN	↑ TT (1) ↑ IR, SPEXIN (2) ↓ LH/FSH, OREXIN (2) ↓ LEPTIN, (1) > (2) BMI, (2 > 1)	[58]
23.	PCOS women (18–35 y)	Canada	Parallel-group-stratified RCT	1 = pulse-based diet (2 pulse meals) 2 = TLC diet (counselled for lifestyle modification) Stratified for metformin 16 weeks	WT, HT, BMI, WC, TG, TC, LDL, HDL, hs-CRP, LH, FSH, HbA1C, SHBG, DHEAS, 17-OHP, TSH, OGTT, Diet, PAL	↓ INS, DBP, TG, TC/HDL,(1) ↑ HDL (1)	[59]
**Calorie-Restricted Diet**							
24.	PCOS women, 18–45 years, BMI 25–35 kg/m^2^ (*n* = 72)	Iran	Double-blinded RCT	1 = LCD + 3 capsule of licorice extract (500 mg licorice+ 36.5 mg glycyrrhizic acid 2 = placebo (cornstarch + LCD) LCD = 1200/1800 kcal, 55/25/30% carb/protein/fat 8 weeks	WT, FM, BMI, FBG, INS, LDL, HDL, TC, TG, HOMA IR, HOMA B	BMI, FM, HOMA IR, TC, TG, LDL, HDL (1)	[60]
25.	PCOS pregnant women, 18–45 years, BMI > 25 kg/m^2^ (*n* = 296)	China	RCT	1 = individualized diet and exercise (1500/1600 kcal, 55/20/25% carbs/protein/fat) 2 = group education	GWG, GDM, PIH, TC, LDL, TG, HDL, FBG, FINS, HOMA IR	↓ GWG (1)	[61]
26.	PCOS overweight/obese women, aged (18–45 years), >24 kg/m^2^ (*n* = 68)	Shenghai, China	RCT (open label)	1 = Dulaglutide + CRD 2 = CRD (1000/1300 kcal, 55/20/30%, carb/protein/fat) 6 months	WT, WC, BMI, FBG, FINS, HbA1C, ALT, AST, TC, TG, HDL, LDL, SUA, SHBG, INSPP, PPG, OGTT, LH, FSH, TT, PRL, FT, AD, DHEAS, FAI, HOMA IR	Shorter time to loss 7% wt loss (1) ↓ HbA1C, PPG (1)	[62]
27.	Overweight PCOS women, aged 16–45 years, BMI > 24 kg/m^2^ (*n* = 72)	China (med)	RCT	1 = MD/LC, *n* = 36, (<20% CHO,100 g/day, ↑ proteins, fats, whole grains, good fats) 2 = LF, *n* = 36, (<30% fat, 40 g/day, ↑ fruits and vegetables,) 12 week	WT, BMI, WC, WHR, INSF, FBG, HOMA IR, menstrual irregularity, QUICKI, TC, TG, LDL, HDL, TT, LH, FSH, PRL, BF%, E2,	↓ WT, BMI, WC, WHR, BF%, INSF, HOMA IR, TG, QUICKI, LDL, (1 > 2) ↓ QUICKI, HOMA IR, FBG, INSF, WT, BMI, WHR, BF%, TT, LH, LH/FSH (1)	[63]
28.	PCOS women aged 13–18 years, BMI ≥ 95 percentile (*n* = 40)	Turkey	RCT (single-blinded)	1 = RESMENA, *n* = 20 (7 meals/d, 40% CHO, 30% protein, 30% Fat) 2 = Control, *n* = 20 (3–5 meals/d, 55% CHO, 15% protein, 30% Fat) 6 months	Diet Quality, TEI, WT, PAL, FM, BF%, FFM, HC, WC, WHR, NC, BMI, FBG, INSF, TC, LDL, HDL, TG, ALT, HbA1C, TT, FT, DHEAS, AD, PRL, 17-OHP, SHBG, IL-6, TNF-α, hs-CRP, FG score, LH/FSH, HOMA IR, QUICKI, FAI, CHO, fiber, omega-3,	↓ TEI, BMI, FM, BF, WT, WC, HC, FFM, (1 > 2) ↑ Diet Quality, meal frequency, omega-3, fiber, SHBG, QUICKI (1) ↓ TT, FT, 17-OHP, LH, LH/FSH, FAI, HbA1c, HOMA-IR, hs-CRP (1)	[64]
29.	Adolescent PCOS, aged 15–17 years (*n* = 40)	Greece (med)	RCT	1 = MD (*n* = 20), individualized dietary intervention 2 = control (*n* = 20) 3 months	WT, BMI, diet quality, anxiety, depression, vitamin D, TG, FM, fiber, Ca	↓ WT (1) > (2) ↑ vitamin D, fiber (1 > 2) ↓ TG, anxiety, FM, Ca, (1) ↑ diet quality (1)	[65]
30.	Obese PCOS women, aged 20–45 years, BMI 30–40 kg/m^2^ (*n* = 48)	Iran	RCT	1 = intervention (*n* = 21), 5 g thylakoid + CRD 2 = placebo (*n* = 23), 5 g cornstarch + CRD 12 week	WT, BMI, HC, WC, FM, FFM, WHR, FBG, INS, NEFA, HOMA IR, HOMA B, QUICKI, LH, FSH, DHEAS, TT, SHBG	↓ WT, WC, FM, HOMA-IR, HOMA-B, QUICKI, INS, TT, (1 > 2) ↑ SHBG (1) ↓ DHEAS (1)	[66]
31.	PCOS women, aged (18–45 years), BMI 30–45 kg/m^2^ (*n* = 40)	UK	RCT (open label)	1 = VLCD (800 increased to 1600 kcal) 2 = moderate-energy-deficit diet 16 weeks	WT, BMI, WC, HC, FBG, FINS, TC, TG, HDL, LDL, HOMA IR, hs-CRP, BP, FAI	↓ FAI, BW, WC, TC, FBG (1) ↑ SHBG (1)	[67]
**Time-Restricted Diet**							
32.	PCOS women aged (18–40 years), BMI > 24 kg/m^2^, (*n* = 18)	China	RCT	6 weeks (1 week stabilizing + 5 week TRF (time-restricted feed)	BW, BMI, WHR, SMM, BFM, BF%, VFA, LH, FSH, LH/FSH, TT, SHBG, FAI, FG, FINS, HOMA IR, TG, TC, LDL, HDL, hs-CRP, ALT, AST, IGF 1, emotional eating, cognitive restraint scale	↓ BMI, BFM, BF%, VFA, TT, FAI, FINS, HOMA IR, hs-CRP ↑ menstrual regularity, SHBG, IGF 1	[68]
**Microbiome-Rich Diet**							
33.	PCOS women, aged 18–45 years (*n* = 40)	Poland	RCT	1 = energy-restricted group 2 = energy-restricted + *Lactobacillus rhamnosus* (50/20/30%, carbs/protein/fat) 20 weeks	WT, WC, FM, BMI, TC, HDL, LDL, TG, Fecal SCFA (acetic, propionic, butyric acid)	↓ WT, BMI, FM, acetic acid, (1 = 2) ↓Butyric acid, LDL (1) > (2) ↓ TC, TG (2) > (1)↑ HDL (2) > (1)	[69]
34.	PCOS women, BMI > 25 kg/m^2^ (*n* = 65)	Poland	RCT (double-blinded, placebo)	1 = LM + placebo 2 = LM + synbiotic (*Bifidobacterium lactis*, *Lactobacillus acidophilus*, *Lactobacillus paracasei*, *Lactobacillus plantarum*, *Lactobacillus salivarius*, *Lactobacillus lactis* + *Inulin* + fructooligosaccharides) LM = 1400/1800 kcal+ 30–40 min walking 3 Months	WT, BMI, FG score, OGTT (30, 60, 90, 120), INS, TT, LH, FSH, SHBG, 17-OHP, DHEAS, BF%, TC, TG, HDL, LDL, WC, WHR, HC	↓ 95% BW, 5% BMI, HC, BF%, (1) ↓ 100% BW, 8% BMI, WC. HC, BF%, TT, TG (2) ↑ DHEAS (2)	[70]
35.	PCOS women, aged (18.5–45 years), BMI 18–35 kg/m^2^	Iran	RCT (triple-blinded)	1 = intervention (2-g of 10^11^ *Bacillus coagulans*, 10^10^ *Lactobacillus rhamnosus*, 10^10^ *Lactobacillus helveticus*, 500 mg fructooligosaccharides, 0.7% natural orange flavoring) 2 = placebo (2 g starch + orange flavor) 12 weeks Stratified by BMI <25 kg/m^2^/≥25 kg/m^2^,	WT, HT, BMI, WC, HC, PCOSQ (emotional, menstrual problem, infertility, body hair, weight, PSS, SF-12 (physical, mental)	↑ PCOSQ (emotional, infertility) (1)	[71]
36.	PCOS women, aged (18–40 years) BMI 17–34 kg/m^2^, IR −1.4–4 (*n* = 60)	Iran	RCT (double-blinded)	1 = intervention (vitamin D, 50,000 IU + 4 freeze-dried strains: *Lactobacillus acidophilus*, *Bifidobacterium bifidum*, *Lactobacillus reuteri*, *Lactobacillus fermentum* (2 × 10^9^ CFU/g each) 2 = placebo (corn oil + starch) 12 weeks (Follow-up at 0, 4, 9, 12 weeks)	WT, BMI, FG score, TT, SHBG, sleep quality, depression, anxiety, 25-OH vitamin D, hs-CRP, TAC, GSH, MDA, mental health	↓ TT, FG score, MDA, hs-CRP, depression, stress, anxiety (1) ↑ TAC, GSH, mental health (1)	[72]
37.	PCOS women, aged (18–40), (*n* = 60)	Iran	RCT (double-blinded, placebo control)	1 = 8 × 10^9^ CFU/day probiotic (*Lactobacillus acidophilus*, *Lactobacillus reuteri*, *Lactobacillus fermentum* and *Bifidobacterium bifidum* (2 × 10^9^ CFU/g each) + 200 μg/day selenium 2 = placebo 12 weeks (follow up at 0, 3, 6, 9, 12 weeks)	WT, BMI, FG score, mental health, stress, anxiety, TT, SHBG, FAI, hs-CRP, TAC, GSH, MDA	↑ Mental health, TAC, GSH ↓ TT, FG score, hs-CRP, stress, anxiety, MDA,	[73]
38.	PCOS women, aged (18–40 years) (*n* = 60)	Iran	RCT (double-blinded, placebo control)	1 = intervention (*Lactobacillus acidophilus*, *Lactobacillus casei* and *Bifidobacterium bifidum* (2 × 10^9^ CFU/g each) + 0.8 g inulin) 2 = placebo 12 weeks (follow up at 3, 6, 9, 12 weeks)	WT, BMI, FG score, FAI, hs-CRP, INS, NO, TAC, GLT, MDA, HOMA IR, FBG, SHBG, METs	↑ SHBG, NO (1) ↓ FG score, FAI, hs-CRP, INS, HOMA IR, TT, MDA	[74]
39.	Obese or overweight PCOS women, aged 20–44 years, BMI 25–44 kg/m^2^	Iran	RCT	1 = synbiotic (*n* = 34), Lactobacillus casei 3 × 10^9^ CFU/g, *Lactobacillus rhamnosus* 7 × 10^9^ CFU/g, *Lactobacillus bulgaricus* 5 × 10^8^ CFU/g, genus Lactobacillus acidophilus 3 × 10^10^ CFU/g, *Bifidobacterium longum* 1 × 10^9^ CFU/g, *Streptococcus thermophilus* 3 × 10^8^ CFU/g, inulin-type prebiotics, FOS 2 = Placebo (*n* = 34), Starch 8 weeks	WT, BMI, WC, HC, WHR, WHtR, PAL, INS, HOMA IR, TG, TC, HDL, LDL, FBG	↓ HOMA IR, INS, WC, WHR, HC, WT, FBG, (1) ↑ BMI, WT (2) ↑ HDL (1)	[75]
40.	Married PCOS women, aged 18–38 years, (*n* = 40)	Iran	RCT	1 = Lactofem plus letrozole (*n* = 20), *Lactobacillus acidophilus* 2 × 10^9^, *Bifidobacterium bifidus* 2 × 10^9^, *Lactobacillus rutri* 2 × 10^9^, *Lactobacillus fermentum* 2 × 10^9^; capsule weight of 500 mg bio-capsule 2 = Only Letrozole (*n* = 20)	BMI, WT, ACNE, FG score, clinical and chemical pregnancy, sexual function, body image, INS, FBG, TT, LH/FSH	↑ Chemical and clinical pregnancy, sexual function, body image (1)	[76]
41.	PCOS women aged (19–37 years)	Iran	RCT (double-blinded)	1 = synbiotic supplement (500 mg of seven strains beneficial bacteria) 2 = placebo (500 mg starch and maltodextrins) 12 weeks	WT, BMI, TG, TC, HDL, LDL, FSH, LH, E2, Progesterone, TT	↑ HDL (1) ↓ LDL (1)	[77]
42.	PCOS women, aged (19–37 years), BMI > 25 kg/m^2^ (*n* = 88)	Iran	RCT (double-blinded, placebo)	1 = intervention (*Lactobacillus acidophilus* 3 × 10^10^(CFU)/g, *Lactobacillus casei* 3 × 10^9^ CFU/g, *Lactobacillus bulgaricus* 5 × 10^8^ CFU/g, *Lactobacillus rhamnosus* 7 × 10^9^ CFU/g, *Bifidobacterium longum* 1 × 10^9^ CFU/g, *Bifidobacterium breve* 2 × 10^10^ CFU/g, *Streptococcus thermophilus* 3 × 10^8^ CFU/g), prebiotic inulin 2 = placebo (starch + maltodextrin) 12 Weeks	WT, BMI, FBG (0, 2 h), HbA1C, hs-CRP, apelin 36, HOMA IR, QUICKI,	↓ apelin 36 (1)	[78]
**Micronutrient supplement**							
43.	PCOS women, aged 18–45 years (*n* = 64)	Iran	RCT	1 = intervention (*n* = 32), magnesium 250 mg/day 2 = control (*n* = 32), placebo 5 g starch 10 weeks	DHEAS, TT, sleep quality, FG score,	↑ DHEAS (1) ↑ Sleep quality, TT (2) ↓ FG score (2)	[79]
44.	Infertile PCOS women, >18 years, (*n* = 48)	Turkey	RCT	1 = micronutrient (*n* = 24) betaine 200 mg, l-cystine 200 mg, chelated zinc 10 mg, niacin (vit. B3) 16 mg, pyridoxine (vit. B6) 1.4 mg, riboflavin (vit. B2) 1.4 mg, 5-methyl-tetrahydrofolate, 400 μg, methylcobalamin 2.5 μg 2 = control (*n* = 24)	Clinical pregnancy, blastocyst rate	Clinical pregnancy 58% (1) Blastocyst rate 55% (1)	[80]
45.	PCOS women, aged 18–45 years, (*n* = 64)	Iran	RCT	1 = intervention (*n* = 32), 250 mg magnesium 2 = placebo (*n* = 32), 10 weeks	Physical functioning, physical health, emotional problem, emotional wellbeing, energy fatigue, QoL, general health, social functioning, acne, alopecia, AUB	↑ Physical functioning, physical health, emotional problem, emotional wellbeing, energy fatigue, QoL, general health, social functioning	[81]
**Essential Amino Acid Supplement**							
46.	Adolescent PCOS women, aged 12–21, BMI > 90 percentile kg/m^2^ (*n* = 21)	Colorado, USA	RCT	1 = intervention, 15 g EAA, 10% histidine, 11% isoleucine, 32% leucine, 16% lysine, 10% phenylalanine, 10% threonine, and 11% valine 2 = placebo 3 months	WT, BMI, TG, AST, TT, HOMA IR, VLDL, PAL, TDE, WC, ALT, SHBG, FFA, HS, SUGAR, Visceral fat, subQ fat, BF%, PCOS-HS index	↓ AST, HS, VLDL, TG, SUGAR (1)	[82]
**DASH Diet**							
47.	Overweight/obese PCOS women, aged 18–40 y, BMI > 25 kg/m^2^ (*n* = 48)	Iran	RCT	1 = DASH diet (52% CHO, 18% proteins, and 30% fats, Na < 2400 mg/day) (*n* = 24) 2 = control group (*n* = 24) 8 weeks	WT, BMI, WC, HC, FBG, INS, HOMA IR, HOMA B, QUICKI, hs-CRP	↑ QUICKI (1) ↓ WC, HC, INS, HOMA IR, hs-CRP (1)	[83]
48.	Overweight or obese PCOS women, aged 20–40 years, BMI 25–40 kg/m^2^ (*n* = 60)	Iran	RCT	1 = DASH Diet (50–55% CHO, 15–20% Proteins, and 25–30% Fats, Na < 2400 mg/day) (*n* = 30) 2 = control group (*n* = 30) 12 week	HT, WT, BMI, WHR, WC, HC, SHBG, FAI, androstenedione, TT, TAC, FM	↓WT, BMI, FM, androstenedione, TT, FAI, (1) ↑ SHBG, TAC (1)	[84]
49.	Overweight/obese PCOS women, Aged 18–40 years, BMI > 25 kg/m^2^ (*n* = 48)	Iran	RCT	1 = DASH diet (52% CHO, 18% proteins, and 30% fats, Na < 2400 mg/day) (*n* = 24) 2 = control group (*n* = 24) 8 weeks	WT, BMI, TG, TC, HDL, LDL, GSH, TAC, VLDL	↑ TAC, GSH (1) ↓ WT, BMI, TG, VLDL, (1)	[85]

* Note: ↑ sign indicate increase and ↓ sign indicate decrease in the outcome mentioned adjacent to it. Parenthesis represents the groups in which significant difference was observed as compared to baseline. (<, =, >) signs indicates the intergroup relationship for the mentioned outcome. Abbreviations: VLCKD (very-low-calorie keto diet), LCD (low-calorie diet), E2 (estradiol), SHBG (sex-hormone-binding globulin), HT (height), FM (fat mass), FFM (fat-free mass), IR (insulin-resistant), DBP (diastolic blood pressure), SBP (systolic blood pressure), GWG (gestational weight gain), GDM (gestational diabetes), PIH (pregnancy-induced hypertension), FBG (fasting blood glucose), ALT (alanine aminotransferase), AST (aspartate aminotransferase), TC (total cholesterol), TG (triglyceride), SUA (serum uric acid), INSPP (insulin postprandial), PPG (postprandial glucose), OGTT (oral glucose tolerance test), PRL (prolactin), FT (free testosterone), AD (androstenedione), DHEAS (dehydroepiandrosterone sulfate), BF% (body fat percentage), TEI (total energy intake), PAL (physical activity level), NC (neck circumference), LDL (low-density lipoprotein), HDL (high-density lipoprotein), 17-OHP (17-hydroxy progesterone), IL-6 (interleukin factor 6), TNF-α (tumor necrotizing factor alpha), FAI (free androgen index), CHO (carbohydrate), Ca (calcium), INS (insulin), NEFA (non-esterified fatty acid), WT (weight), BMI (body mass index), WC (waist circumference), HC (hip circumference), WHR (waist hip ratio), MD (Mediterranean diet), RESMENA (metabolic syndrome reduction in Navarra), LC (low-carbohydrate), CRD (calorie-restricted diet), HSDF (hypocaloric standard diet with fennel), HHPF (hypocaloric high-protein diet with fennel), HSDP (hypocaloric standard diet with placebo), HHPP (hypocaloric high-protein diet with placebo), INSF (insulin fasting), HOMA B (homeostasis model assessment of β-cell), QUICKI (quantitative insulin sensitivity check index), VLDL (very-low-density lipoprotein), SFA (saturated fatty acids), hs-CRP (high-sensitivity C-reactive protein), NO (nitric oxide), TAC (total antioxidant capacity), GSH (glutathione), MDA (malondialdehyde), TT (total testosterone), LGL (low glycemic load), HGL (high glycemic load), UK (United Kingdom), USA (United States of America), HbA1C (haemoglobinA1c), FG score (Ferryman–Gallwey score), HOMA IR (homeostasis model assessment of index), LH (luteinizing hormone), FSH (follicle-stimulating hormone), LF (low-fat) WTP (whole grain, traditional Chinese, prebiotics), CAT (catalase activity), EPA (eicosapentaenoic acid), DHA (docosahexaenoic acid), TSH (thyroid-stimulating hormone), GGT (gamma-glutamyl transferase), BP (blood pressure), BIL (billubirin), MN (micronutrient), NAC (*N-acetyl* cysteine), SAMe (S-Adenosylmethionine), B6 (vitamin b6), ALA (Alpha-lipoic acid), OC (oral contraceptive), ET (endometrium thickness), LPF (lipopolysaccharide), BNDF (brain-derived neurotrophic factor), OS (oxidant stress), S 100B (S100 calcium-binding protein B), FTI (free testosterone index), QoL (quality of life), CoQ10 (co-enzyme Q10), IU (international unit), ALP (alkaline phosphatase), BUN (blood urea nitrogen), MUFA (monounsaturated fatty acid), fecal SCFA (short-chain fatty acid), LM (lifestyle modification), PCOSQ (PCOS quality of life), PSS (perceived stress score), SF-12 (short form), CFU (colony forming units), MET (metabolic equivalent), WHR (waist–height ratio), AMH (anti-mullerian hormone), P (progesterone), O3 (omega-3), TDE (total dietary intake), HS (hepatic stenosis), subQ fat (subcutaneous fat), PCOS-HS (health survey) index, AUB (abnormal uterine bleeding).

**Table 2:** List of studies investigating the effects of physical activity (exercise) PCOS outcomes.

S. No	Participant Details (*n* = Sample Size)	Location	Study Design	Intervention and Duration	Control	Outcomes Studied	Major Findings *	References
1.	Women with PCOS, age = 18 to 39 years, BMI = 18–39.9 kg/m^2^ (*n* = 87)	Brazil	RCT	1 = CAT (*n* = 28) 2= IAT (*n* = 29) Treadmill exercises, 3 times/week for 16 weeks At 60–90% HRmax Duration = 30 min in first week and 50 min in last week	3 = no intervention (*n* = 30)	FSH, LH, TSH, SHBG, 17-OHP, Pr, estradiol, H, CRP, HDL, C, TG, LDL, FAI, BMI, WC, HC, WHR, BF, HRmax, TL	↓ HC, HR, T, C, LDL, BF (1 > 2) ↓ WHR, FAI (2 > 1) ↓WC (1 = 2 = 3)	[147]
2.	Women with PCOS, age = 18 to 39 years, (*n* = 75)	Brazil	RCT	1 = MICT = 50 min, 3 times/week (*n* = 25) 2 = HIIT = 35–45 min (*n* = 25) Duration = 16 Weeks	3 = no intervention (*n* = 25)	I, C, BMI, VO_2_ peak, VO_2_, VCO_2_, HR, SBP, DBP, MBP, ECG, HRV, BPV, BRS	↓ HR, T (1 = 2) ↑ VO_2_peak (1 = 2)	[148]
3.	Women with PCOS, age = 25 to 35 years, BMI = 23.6 ± 3.5 kg/m^2^, (*n* = 40)	Egypt	RCT	1 = AEM = treadmill walking, 3 sessions/weeks + 1500 mg Metformin for 12 weeks, (*n* = 20) 2 = M = 1500 mg Metformin (*n* = 20) Duration = 12 weeks	--	IL-6, CRP, TNF-α, TVS, BMI, MFG score	↓ IL-6, CRP, TNF-α (1 > 2)	[149]
4.	Women with PCOS, age = 18–40 years, (*n* = 47)	Canada	Pilot RCT	1 = HIIT = BMI < 28 kg/m^2^ = 10 cycles of 30 s at high intensity (90% of HRR), (*n* = 16) 2 = CAET = BMI > 28 kg/m^2^ = 40 min of moderate-intensity aerobic exercise (50–60% HRR), (*n* = 14) Duration = 40 min	3 = no intervention, (*n* = 17)	BMI, Glu, C, HDL, TG, ALT, GGT, LDL, VO2 max	↓ BMI (2 > 3), ↓ LDL (1 > 2), ↑ HDL (1 > 3)	[150]
5.	Women with PCOS, (*n* = 43)	Australia	RCT	1 = DO, 6000 KJ/day energy-restricted high-protein meal plan (*n* = 13) 2 = DA, diet + walking/jogging 5 times/week for 20–25 min (*n* = 11) 3 = DC, diet + jogging 3 times/week, strength training 2 times/week (*n* = 19) Duration = 20 weeks	--	EBBSscore, BMI, VO_2_ peak, PCOSQ, CES-D	↑ VO_2_max (2 > 3) ↓ weight and ↓ depressive symptoms (2 > 3)	[151]
6.	Women with PCOS, (*n* = 31)	Norway	Pilot RCT	1 = HIIT = BMI > 27 kg/m^2^, HIT 2 sessions 4 times 4 min at 90–95% of HRmax) (*n* = 10) 2 = ST = < 27 kg/m^2^ 8 dynamic strength drills with resistance of 75% of 1 RM (*n* = 11) Duration = 10 weeks	3 = 150 min of MIE (*n* = 10) Duration = 10 weeks	HOMA-IR, G, C, HDL, LDL, TG, CRP, I, adiponectin, TG, L, T, H, AMH, SHBG, DHEAS, FMD, W, BC, VF, VO_2_max, FMD, FAI, MFG score	↑ HOMA-IR, HDL, FMD (1 > 2) ↓ AMH, FAI (2 > 1)	[152]
7.	Women with PCOS, age = 18 to 40 years, (*n* = 45)	Iran	RCT	1= HIIT 3 repetitions of sprint running for 30 secs-by 30 Secs of slow running and 5 min of dynamic stretching, (*n* = 15) 2= COM (RT + MICT), 30 to 40 min, RT (3 sets, 50–70% of 1 RM), Duration = 8 weeks (*n* = 15)	3 = no intervention (*n* = 15)	W, BMI, WHR, FP, VAT, VO_2_max, MSFT	↓ W, BMI, WHR, FP, VAT (1 = 2) ↑ in VO_2_max (1 = 2)	[153]
8.	PCOS overweight/obese women, age = 18–34 years, BMI = 25–39.9 kg/m^2^ (*n* = 27)	Brazil	RCT	1 = 14 = AE warm up = 5 min aerobic exercise = 40 min cool down = 5 min Duration = 3 times/week for 16 weeks	2 = no intervention (*n* = 13)	HRQL, VO_2_max, W, H, BP, WC, OGTT, HDL, LDL, TNF-α, IL-6, CRP, HOMA-IR, MFG, DHES, LH, FSH, HRmax, CRF, C, SBP, DBP	↑ CRF and HRQL (1) ↓ BMI, WC, SBP, DBP, C (1)	[154]
9.	Women with PCOS age = 18–40 years (*n* = 28)	Iran	RCT	1 = 14 = HIIT Performed at 100–110 MAV, 4–6 sets, 4 laps Duration = 3 times/week for 8 weeks	2 = no intervention (*n* = 14)	VAI, Glu, HDL, AIP, H, CRP, W, BMI, FP, WHR, VAT, VO2max, I, HOMA-IR, LDL, C, HDL, TG.	↓ BMI, WHR, VF, I, IR, LDL, AIP, C, CO (1)	[155]
10.	Women with PCOS Age = 14–18 years BMI = 28.52 kg/m^2^) (*n* = 40)	Iran	RCT	1 = 20 = AE Warm up = 10 min, aerobic workout = 40 min, cool down = 10 min Duration = 3 times/week for 12 weeks	2 = no intervention (*n* = 20)	W, BMI, T, Pr, E, BM, BMI, C, TG, LDL, HDL	↓ T, Pr, E, BM, BMI, C, TG, LDL (1) ↑ HDL (1)	[156]
11.	Women with PCOS, age = 18–45 years BMI =< or >= 27 kgm^2^ (*n* = 64)	Australia Norway	Two centre RCT	1 = HV-HIIT (*n* = 20) 2 = LV-HIIT (*n* = 21) Duration = 3 times/week for 12 weeks	3 = no intervention (*n* = 23)	PCOSQ, FG score, HRmax, VO_2_max, OGTT, HOMA-IR, C, LDL, HDL, TG, Pr, T, AMH, SHBG, I, WC, HC, FP, BP, pregnancy rate	↑ Pregnancy rate (2 > 3) Menstrual frequency = no difference = 1 = 2 = 3	[157]
12.	Women with PCOS, age = 18–39 years (*n* = 87)	Brazil	RCT	1 = CAT (*n* = 28) 2 = IAT (*n* = 29) Treadmill exercises, 3 times/week for 16 weeks At 60–90% HRmax Duration = 30 min in first week and 50 min in last week	3 = no intervention (*n* = 30)	FSH, LH, TSH, SHBG, 17-OHP, Pr, estradiol, H, CRP, HDL, C, TG, LDL, FAI, BMI, WC, HC, WHR, BF, HRmax, FP, TFP, LFP, TGM	↓ WC, HP, C, LDL, T (1) ↓ FAI (2) ↑ WC, FP, TFP, LFP, TGM (3)	[158]
13.	Women with PCOS, aged 18–40 years, 18.5–40 kg/m^2^ (*n* = 23)	Brazil	RCT	1 = Intervention (*n* = 12), HIIT 40–60 min, 3 days/week followed by detraining 30 days 12 weeks	2 = Control (*n* = 11)	W, BMI, WC, HC, WHR, QOL, anxiety, depression, FG score, FBG, OGTT, TC, HDL, LDL, TG, total fat, trunk fat, gynoid, android	↑ QOL (1) ↓ Anxiety, Depression (1)	[159]

* Note: ↑ sign indicate increase and ↓ sign indicate decrease in the outcome mentioned adjacent to it. Parenthesis represents the groups in which significant difference was observed as compared to baseline. (<, =, >) signs indicates the intergroup relationship for the mentioned outcome. Abbreviations: group (G), control group (CG), minutes (mins), weight (W), height (H), aerobic exercise (AE), continuous aerobic training (CAT), intermittent aerobic training (IAT), aerobic exercise group (AEM), metformin group (M), continuous aerobic exercise training (CAET), heart rate reserve (HRR), diet only (DO), diet and aerobic exercise (DA), diet and combined aerobic resistance exercise (DC), high-intensity training (HIT) high-intensity interval training (HIIT),physical exercise (PE), moderate intensity (MI), insulin resistance (HOMA-IR), strength training (ST), between (b/w), heart rate maximum (HRmax), anti-Müllarian hormone (AMH), insulin resistance (IR), inulin fasting (I-F), insulin postprandial (I-PP) flow-mediated vasodilation (FMD), free androgen index (FAI), clomiphene citrate (CC), moderate-intensity continuous training (MICT), depression anxiety stress scales (DASS-21), 36–item short-form health survey (SF-36), polycystic ovary syndrome questionnaire (PCOSQ), body composition (BC), body fat (BF), maximal oxygen consumption (VO2 max), peak heart rate (HRpeak), insulin sensitivity index (ISI), metabolic equivalent (MET), depression (D), anxiety (A), stress score (SS), physical functioning (PF), general health (GH), combined resistance training (CRT), moderate-intensity interval continuous training (MICT), multi stage fitness test (MSFT), fat percentage (FP), visceral adipose tissue (VAT), waist to hip ratio (WHR), hip circumference (HC), homocysteine (H), leptin (L), visceral fat (VF), modified Ferriman–Gallwey score (MFG score), waist (W), waist circumference (WC), luteinizing hormone (LH), follicle-stimulating hormone (FSH), thyroid-stimulating hormone (TSH), prolactin (Pr), testosterone (T), dehydroepiandrosterone sulfate (DHEAS), sex-hormone-binding globulin (SHBG), insulin (I), glucose (Glu), high-density lipoprotein (HDL), low-density lipoprotein (LDL), total cholesterol (C), triglycerides (TG), serum glutamic oxloacetic transaminase (SGOT), C-reactive protein (CRP), telomere length (TL), transvaginal scan (TVS), hyperandrogenism (HA), 75-g oral glucose tolerance test (OGTT), cortisol (CO), androstenedione (A), muscle biopsy (MB), body fat mass (BFM), body mass (BM), estradiol (ES), fat-free mass (FFM), repetition maximum (RM), moderate-intensity exercise (MIE), oral contraceptive pill (OCP), lifestyle modification (LM), calorie restriction (CR), meal replacement (MR), antiobesity medication (AOM), behavioral modification (BM), physical activity (PA), systolic blood pressure (SBP), diastolic blood pressure (DBP), mean blood pressure (MBP), electrocardiographic (ECG), heart rate (HR), blood pressure variability (BPV), baroreflex sensitivity (BRS), ventilation of carbon dioxide production (VCO_2_), volume of oxygen (VO_2_), moderate-intensity exercise (MIE), quality of life (QOL), ovarian volume (OV), antral follicle count (AFC), cardio respiratory fitness (CRF), atherogenic index of plasma (AIP), maximum aerobic velocity (MAV), estrogen (E), 17-hydroxyprogesterone (OHP), low-volume–high-intensity interval training (LV–HIIT), high-volume–high-intensity interval training (HV-HIIT), trunk fat percentage (TFP), leg fat percentage (LFP), total gynoid mass (TGP),free androgen index (FAI), quality of life SF–36 (QOL SF-36), health-related quality of life (HRQL), resistance training(RT), baroreflex sensitivity (BRS), and mean blood pressure (MBP), peak oxygen uptake (VO2 peak), body mass index (BMI), blood pressure (BP), Ferriman–Gallwey score (FG score), glucose tolerance test (GTT), Exercise Benefits/Barriers Scale (EBBS), Centre for Epidemiologic Studies Depression Scale (CES-D), visceral adiposity index (VAI), interleukin-6 (IL6), tumor necrosis factor (TNF-α), alanine transminase (ALT), heart rate variability (HRV), combined (COM), fasting blood glucose (FBG).

**Table 3:** List of studies investigating the effects of behavioral and education modules on PCOS management.

S. No.	Participant Details (*n* = Sample Size)	Location	Study Design	Intervention and Duration	Control	Outcomes Studied	Major Findings *	Reference
1.	Women with PCOS, age => 18 years BMI => 25 kg/m^2^ (*n* = 122)	China	RCT	1 = TTM (*n* = 61) expert consultation provided—online chat assessment and recording of diet and exercise (consisting of PAG, AG, MG)	2 = routine care, general advice on diet and exercise (*n* = 61)	SDS, SAS, WC, BMI, SDC	↓ BMI, WC, SAS, SDS, anxiety, depression (1) ↑ exercise, diet adherence (1)	[171]
2.	Women with PCOS, (*n* = 84)	Iran	RCT	1 = Counselling + CBT (*n* = 42) Duration = 8 sessions of 60–90 min/week	2 = routine care (*n* = 42)	SDOBQ, STAI, BDI, PCOSQ,	↓ anxiety, depression (1) ↑ QOL (1)	[172]
3.	Women with PCOS Age = 18–38 years BMI => 25 kg/m^2^ (*n* = 183)	Netherlands	RCT	1 = LI with SMS, maintaining active lifestyle by exercising and developing healthy dietary habits (*n* = 60) 20 CBT sessions with SMS 2 = LI without SMS = 20 CBT sessions without SMS (*n* = 63) Duration = 12 months	3 = CAU = unstructured consultation by physician (*n* = 60)	DEBQ, EDEQ	Improved EDEQ scores (1 = 2)	[173]
4.	Women with PCOS Age = 18–45 years (*n* = 13)	Australia	RCT	1 = SIT = 3.5 h (*n* = 7) 2 = sitting interrupted by 3 min bouts of SRA every 30 min (*n* = 6) Duration = 3.5 h	--	FMD, resting BP, resting FSR	↑ mean FSR, BP (2 > 1)	[174]
5.	Women with PCOS, aged (18–42 years), (*n* = 52)	Iran	RCT (parallel, 3 stage design) 3 stage = pre, post (immediately), Follow-up (after 1 month)	1 = Intervention (ACT-8 sessions of 90 min/week) 1 month	2 = control	Body image concerns, self-esteem	↓ body image concern (at all 3 stages (1) ↑ self-esteem (at all stages) (1)	[175]
6.	PCOS women, aged (18–49 years) (*n* = 85)	Malaysia	RCT (single-blinded)	1 = Intervention (info regarding PCOS, MS, Healthy lifestyle, nutrition, physical activity) Diet—8 sessions (30 min/session) Exercise—32 session (45 min/session) 2 session on motivation 1 session on stress 2 session of self-discipline 2 session on sleep and rest 6 Months	2 = control (usual lifestyle modification)	KAP of diet, KAP of physical activity, SBP, DBP, EAT-26, TFEQ –R18, IPAQ	↑ knowledge of nutrition, IPAQ	[176]
7.	PCOS women, aged (*n* = 70)	Iran	RCT (double-blinded)	1 = 5As Assess—knowledge, beliefs, lifestyle Advise—lifestyle, nutrition, physical activity Agree—identify behavioral goal, changing behavior and implementing practical plan Assist—discuss barriers to implementation of lifestyle Arrange—progress of diet and exercise was followed via call 4 sessions of 40–45 min 3 months	2 = without 5As model	psychological symptoms, somatization, interpersonal sensitivity, anxiety, depression, hostility, obsessive–compulsive disorders	↑ psychological symptoms, somatization, interpersonal sensitivity, anxiety, and hostility decreased (1)	[168]
8.	Infertile women with PCOS, aged 15–40 years (*n* = 60)	Iran	RCT	1 = intervention, *n* = 30 (motivational interview 1/week, five sessions) 2 MONTH	2 = control, *n* = 30 (routine care)	health responsibility, physical activity, nutrition, spiritual growth, interpersonal relations, stress management, QOL	↑ health responsibility, physical activity, nutrition, spiritual growth, interpersonal relations (1)	[177]
9.	Women with PCOS, aged (18–40 years), BMI > 27 kg/m^2^ (*n* = 68)	Sweden	RCT	1 = intervention (behavior modification, formal course, group meetings 3 times/month, with individualized coaching) 4 months	2 = control (minimal intervention, healthy lifestyle recommendation)	HT, BW, WT, WHR, menstrual function, BF%, LBM, trunk fat, SHBG, HOMA IR, ET, TT, FAI	↑ Menstrual function (59%), ET (1) ↓ BW (2.1%), BF%, Trunk fat (1) ↓ TT, FAI (2)	[167]
10.	Women with PCOS, aged (14–23 years) (*n* = 37)	Central Texas	RCT (pre–post)	1 = mindfulness training program (medication adherence, nutrition, physical activity, sleep) Session of lifestyle intervention via focus group meetings 5 week	-	psychological distress, mindfulness, physical activity strategies, nutrition, exercise self-efficacy	↑ nutrition self-efficacy, physical activity strategies, physical activity self-efficacy (1)	[170]
11.	Women with PCOS, age = 18–38 years BMI => 25 kg/m^2^ (*n* = 183)	Netherlands	RCT	1 = LI with SMS, maintaining active lifestyle by exercising and developing healthy dietary habits(*n* = 60) 20 CBT sessions with SMS 2 = LI without SMS = 20 CBT sessions without SMS (*n* = 63) Duration = 12 months	3 = CAU = unstructured consultation by physician (*n* = 60)	IPAQ, PAR-Q	↑ IPAQ (1 > 3)	[178]
12.	Women with PCOS age = 18–38 years BMI => 25 kg/m^2^ (*n* = 183)	Netherlands	RCT	1 = LI with SMS, maintaining active lifestyle by exercising and developing healthy dietary habits (*n* = 60) 20 CBT sessions with SMS 2 = LI without SMS = 20 CBT sessions without SMS (*n* = 63) Duration = 12 months	3 = CAU = unstructured consultation by physician (*n* = 60)	HOMA-IR, BP, WC, Glu, I, cMetS z-score	↓cMetS z-score (1 > 3)	[179]
13.	Women with PCOS age = 18–45 years (*n* = 66)	Iran	RCT	1 = TTM–we conducted chat-based motivational interviews, diet and exercise diary (*n* = 35) Duration = 6 months	2 = unstructured advised about healthy behavior (*n* = 31)	WC, BMI, SDC, HPLP-II	↑HPLP-II (1) ↓ WC, BMI (1)	[180]
14.	Women with PCOS, age = 18–40 years BMI => 25 kg/m^2^ (*n* = 28)	Korea	RCT	1 = LM-mobile application–calculation of calories, exercise time, hirsutism and acne scale (*n* = 14) Duration = 12 weeks	2 = maintain usual lifestyle (*n* = 14)	FSH, LH, SHBG, DHEAS, ES, T, FG score, GAGS, K-CESD, I F and PP	↓ weight, depressive symptoms, I PP, hirsutism (1 > 2)	[181]

* Note: ↑ sign indicate increase and ↓ sign indicate decrease in the outcome mentioned adjacent to it. Parenthesis represents the groups in which significant difference was observed as compared to baseline. (<, =, >) signs indicates the intergroup relationship for the mentioned outcome. Abbreviations: cognitive behavioral therapy (CBT), self-rating depression scale (SDS), self-rating anxiety scale (SAS), waist circumference (WC), body mass index (BMI), sociodemographic characteristics (SDC), sociodemographics and obstetric characteristics questionnaire (SDOBQ), Spielberger state-trait anxiety inventory (STAI), beck depression inventory (BDI), quality of life questionnaire for women with polycystic ovary syndrome (PCOSQ), quality of life (QOL), lifestyle intervention with additional short message service (LI with SMS), care as usual (CAU), Dutch eating behavior questionnaire (DEBQ), eating disorder examination questionnaire (EDEQ), pre-action group (PAG), action group (AG), maintenance group (MG), uninterrupted sitting (SIT), simple resistance activities (SRA), flow-mediated vasodilation (FMD), resting femoral shear rate (FSR), resting blood pressure (resting BP), lifestyle intervention with additional short message service (LSI with SMS), international physical activity questionnaire (IPAQ), physical activity readiness questionnaire (PAR-Q), metabolic syndrome (MetS), MetS severity z-score (cMetS z-score), sociodemographic characteristics (SDC), intervention group (IG), health-promoting lifestyle profile (HPLP)-II, self-efficacy scale for chronic disease (SECD6), transtheoretical model (TTM), aerobic exercise (AE), intertrochanteric section modulus (ISC), estradiol (ES), bone mineral density (BMD), bone mineral content (BMC), fat percentage (FP), testosterone (T), global acne grading system (GAGS), Korean version of the epidemiological studies depression scale (K-CESD), lifestyle modification (LM), resting femoral shear rate (resting FSR), resting blood pressure (resting BP), glucose (Glu), insulin (I), Ferriman–Gallwey score (MFG score), dehydroepiandrosterone sulfate (DHEAS), inulin fasting (I-F), insulin postprandial (I-PP), luteinizing hormone (LH), follicle-stimulating hormone (FSH), sex-hormone-binding globulin (SHBG), therapeutic lifestyle changes (TLC), knowledge attitude practice (KAP), systolic blood pressure (SBP), diastolic blood pressure (DBP), eating attitude test (EAT-26), three factor eating questionnaire (TFEQ – R18), height (HT), weight (WT), waist hip ratio (WHR), body fat % (BF%), lean body mass (LBM), insulin resistance (HOMA-IR), endometrial thickness (ET), total testosterone (TT), free androgen index (FAI), ACT (Acceptance and commitment therapy, PCOS (Polycystic Ovarian Syndrome), MS (Metabolic Syndrome).
